# Quality of Life and Associated Factors among Primary Care Asian Patients with Type 2 Diabetes Mellitus

**DOI:** 10.3390/ijerph16193561

**Published:** 2019-09-23

**Authors:** Hardesh Dhillon, Rusli Bin Nordin, Amutha Ramadas

**Affiliations:** 1 Barwon Health, University Hospital Geelong, Geelong, Victoria 3220, Australia; hardeshdhillon92@gmail.com; 2 Jeffrey Cheah School of Medicine and Health Sciences, Monash University Malaysia, Bandar Sunway, Selangor 47500, Malaysia; 3 School of Medicine, Faculty of Health and Medical Sciences, Taylor’s University, Subang Jaya, Selangor 47500, Malaysia; rusli.nordin@taylors.edu.my

**Keywords:** quality of life, type 2 diabetes mellitus, medication adherence, psychosocial well-being, diabetes complications

## Abstract

Diabetes complications, medication adherence, and psychosocial well-being have been associated with quality of life (QOL) among several Western and Asian populations with diabetes, however, there is little evidence substantiating these relationships among Malaysia’s unique and diverse population. Therefore, a cross-sectional study was conducted in a Malaysian public primary care clinic among 150 patients diagnosed with type 2 diabetes mellitus (T2DM). Structured and validated questionnaires were used to investigate the associations between demographic, clinical, and psychological factors with QOL of the study participants. Approximately three-quarters of patients had a good-excellent QOL. Diabetes-related variables that were significantly associated with poor QOL scores included insulin containing treatment regimens, poor glycemic control, inactive lifestyle, retinopathy, neuropathy, abnormal psychosocial well-being, higher diabetes complication severity, and nonadherence (*p* < 0.05). The main predictors of a good-excellent QOL were HbA1c ≤ 6.5% (aOR = 20.78, 95% CI = 2.5175.9, *p* = 0.005), normal anxiety levels (aOR = 5.73, 95% CI = 1.8–18.5, *p* = 0.004), medication adherence (aOR = 3.35, 95% CI = 1.3–8.7, *p* = 0.012), and an aDCSI score of one and two as compared to those greater than or equal to four (aOR = 7.78, 95% CI = 1.5–39.2, *p* = 0.013 and aOR = 8.23, 95% CI = 2.1–32.8, *p* = 0.003), respectively. Medication adherence has also been found to be an effect modifier of relationships between HbA1c, depression, anxiety, disease severity, and QOL. These predictors of QOL are important factors to consider when managing patients with T2DM.

## 1. Introduction

Diabetes mellitus (DM) is a group of metabolic disorders of various causes that are marked by hyperglycemia. Type 2 diabetes mellitus (T2DM) is the most common form of diabetes which accounts for approximately 80% of all diabetes cases after the age of 30 [1]. Diabetes is an increasingly important medical and public health issue. Despite medical advances in the field of diabetes, the prevalence of T2DM continues to rise globally. The International Diabetes Federation (IDF), in 2015, reported that approximately 415 million adults were affected by DM among which 20% were Southeast Asians. These numbers are projected to rise to 642 million by 2040 [2]. In Malaysia, the prevalence of T2DM, according to the National Health and Morbidity Survey (NHMS), has gone up from 6.3% in 1986 to 17.5% in 2015 [3,4].

The physical and psychosocial impact of T2DM has led to a growing healthcare burden globally. Chronic hyperglycemia, which is characteristic of diabetes, results in physical injury to several organs within the human body. The National Health and Nutrition Examination Survey (NHNES), in 1998, reported the prevalence of myocardial infarction (9.8%), stroke (6.8%), foot problems (22.9%), chronic kidney disease (27.8%), and eye problems (18.9%) among T2DM patients [5]. Glycemic control through medication adherence (MA) has been established as a vital factor for reducing the risk of complications among T2DM patients [6]. Despite the strong evidence, MA remains poor among DM patients, ranging from 36% to 85% [7]. A review by Asche et al., in 2011, reported that only two studies have evaluated the relationship between MA and QOL [8]. The first was performed on 238 patients with the WHO Quality of Life instrument (WHOQOL-100) to assess the QOL and MA using an indirect pill count method. This study found no association between QOL and MA. However, it was limited by its unreliable method of assessing MA through the pill count method from home visits. This method was more likely to influence medication-taking behavior as well causing an overestimation of the actual results [9]. The second study looked at 766 adults with T2DM. It was aimed at highlighting the relationship between medication underuse and health outcomes. One aspect of the study showed medication underuse was associated with lower physical and mental functioning scores based on the 12-item Short Form Health Survey (SF-12) questionnaire. Unfortunately, the actual magnitude of nonadherence was not assessed in this study as the definition for this was rather narrow [10].

A recent local study noted that almost half of Malaysian patients with T2DM were nonadherent to their medication regimen [11]. In Malaysia, a recent cross-sectional study on 700 patients using the Morisky Medication Adherence Scale (MMAS) was carried out to determine the link between MA and QOL among T2DM patients. The total MMAS score correlated modestly with QOL. There was a highly significant association between MA and the environmental component of the QOL scores [12].

Emotional stressors associated with having T2DM often negatively affect a person’s mental and social well-being. There are several disease related factors that have a notable effect on the psychological well-being of T2DM patients. These include, the stress from being diagnosed with a chronic condition, the cost of managing the T2DM, the impact of diabetes related complications, and medication side effects [13]. In 2008, among 85,088 people who participated in the World Mental Health Survey, adults with diabetes had higher rates of depressive and anxiety disorders as compared with those without diabetes [14].

In the last decade, clinicians and researcher have started to recognize the importance of assessing quality of life (QOL) in the management of chronic conditions such as T2DM. Several studies have shown that patients with T2DM have a poorer QOL as compared with those without T2DM [15,16]. Diabetes complications and severity, MA and psychosocial well-being are three factors that have been described to play a major role in the complex relationship between T2DM and QOL.

Microvascular and macrovascular complications and disease severity of diabetes have been declared by many studies as a root cause for poor QOL [17,18]. The results of a recent cross-sectional study in Malaysia showed that complications such as sexual dysfunction, retinopathy, and nephropathy were associated with significantly lower QOL scores [19]. Poor MA has also been linked to having a poorer QOL [10]. A recent study among people with T2DM in Malaysia demonstrated a positive correlation between MA and improved health-related QOL [13].

Higher rates of depression and anxiety have been observed among T2DM patients. Via several bio-behavioral effects, these psychological disorders among T2DM patients result in poorer QOL [16]. A meta-analysis by Egede et al. consistently over a 12-year period, demonstrated a poorer QOL among patients with T2DM who were depressed [20]. A similar trend has also been observed among DM patients with abnormal anxiety levels [21]. 

Despite a growing appreciation for QOL, there are limited studies exploring these complex associations among Malaysians with T2DM. Recently, Goh et al. developed the Asian Diabetes Quality of Life (AsianDQOL) questionnaire, a diabetes-specific questionnaire that demonstrated a high reliability and validity when used among Malaysia’s ethnically diverse population [22]. The questionnaire has high reliability and strong validity when used among Asian people, which originates from features that Asians perceive differently from the Western world such as meal preferences and sexual taboo and these were given consideration in the development of the questionnaire. In 2012, Chang et al. [23] developed the Adapted Diabetes Complication and Severity Index (aDCSI) that aggregated diabetic complications according to severity based on their ability to predict hospitalization and mortality. With the advent of this instrument, relationships between an aggregation of complications and other factors could be established, instead of analyzing individual complication. In addition, different methods have been employed to assess MA over the years. Among them was the Malaysian Medication Adherence Scale (MALMAS), which demonstrated a high sensitivity when used among Malaysians with T2DM [24]. The 21-item Depression Anxiety and Stress Scale (DASS-21) is an assessment tool that has high reliability and strong validity which can not only assess depressive symptoms but also anxiety and stress symptoms [25,26]. In 2007, this questionnaire was translated into local language, Bahasa Malaysia, to suit the local population of Malaysia. This translated version showed strong validity and high reliability with good psychometric properties [27].

Using these four instruments, this study aimed to fill the research gap in Malaysia, by investigating the following three possible predictors and their relationship with QOL among Malaysian T2DM patients: complications and disease severity, MA, and psychosocial well-being. By establishing these relationships, we hope this study contributes to a better and more holistic approach of managing T2DM.

## 2. Materials and Methods 

### 2.1. Study Design and Sampling

This was a cross-sectional study carried out at the Tanglin Health Clinic, an urban government primary care clinic in Malaysia. From March 2017 through April 2017, T2DM adults who presented to the clinic for follow up were invited to participate in the study. The inclusion criteria were as follows: diagnosis of T2DM, age >18 years, Malaysian citizen, and consent to participate. The exclusion criteria were as follows: pregnant female and having an illness which could affect QOL such as cancer, liver disease, and end stage renal disease (ESRD). The population analyzed was a multiethnic group consisting of Malays, Indians, and Chinese T2DM patients who presented to the diabetes clinic for their follow up with their respective doctors. 

The sample size was based on criterion by Green [28], whereby the minimum sample size = 104 + p, where p = estimated number of maximum independent variables. Hence, the minimum sample size for an estimated 20 independent factors and including an estimated additional 20% for dropouts or incomplete data was 149 subjects. Convenience sampling was used to recruit 150 study participants who fulfilled the inclusion and exclusion criteria.

### 2.2. Procedure and Measurements

Patients who attended the clinic had their anthropometric measurements taken and recent blood tests reviewed by the nursing staff prior to receiving an appointment number to see their respective doctors. Following the assessments by the nursing staff, T2DM patients who met the inclusion criteria were identified and invited to participate in the study while waiting for consultation with their respective doctors. 

These selected patients were provided with an explanatory statement and consent form. All participants who had consented were provided with a questionnaire that included seven separate sections: sociodemographic characteristics, anthropometric markers, lifestyle behavior, psychosocial well-being, medication adherence, diabetes complications, and quality of life. Anthropometric details and the section on diabetes complications were completed by the investigator based on the clinical notes and patients’ verbal history. The remaining sections of the questionnaire were completed by participants without assistance from the investigator. 

All parts of the questionnaire, except for the QOL section, were administered in Malay or English depending on the patients’ preference. The AsianDQOL section of the questionnaire was only administered in Malay [20]. This was done to ensure standardization as scoring differed according to the language the questionnaire was administered. The five domains assessed by the QOL questionnaire included energy, memory, diet, sex, and finance. All items were scored on a Likert scale with a higher score correlating with a better QOL.

The DASS-21 and MALMAS questionnaire were used to evaluate psychosocial well-being and assess MA among participants involved, respectively [24,25]. As per the MALMAS questionnaire, those with a sum score of <6 were categorized as nonadherent while those with a score of ≥6 were categorized as adherent. Normal categorization of depression, anxiety, and stress scores were based on the DASS-21 scale. Participants within the mild to extremely severe subscales were combined and categorized as abnormal. Information on their types of medications and supplements, duration of diabetes, and hospital follow-up locations were also obtained. The participants’ level of physical activity was evaluated using the short International Physical Activity Questionnaire (IPAQ) scoring system [29].

Information regarding diabetes complications were obtained from the participants’ clinical notes. All information was corroborated with the participants’ history to ensure accuracy. Three monthly assessments for peripheral neuropathy, peripheral vascular disease, and foot ulcers along with yearly retinopathy and nephropathy screening were conducted and documented at the primary care clinic. Information regarding history of strokes and MIs was obtained from the participants’ diabetes follow-up book. This information was used to complete the aDCSI section of the questionnaire [23].

### 2.3. Statistical Analysis

All data were analyzed using the IBM^®^ SPSS^®^ Statistics 23.0 (IBM Corporation, Armonk, NY, USA). Normality of continuous data was determined using the Kolmogorov–Smirnov test. Continuous data was analyzed and reported as means and standard deviations (SDs) (normally distributed) or medians and interquartile ranges (IQRs) (not normally distributed), whereas categorical data was presented as frequencies and percentages. Differences in proportion between categorical variables and QOL categorical scores were tested using the Chi-square test. Univariate analysis was initially performed between QOL scores and sociodemographic factors and other variables. AsianDQOL mean scores were compared between group using independent *t*-test (2 groups) and one-way ANOVA (≥3 groups). The relationships between the significant independent variables and good-excellent QOL were assessed using multiple binary logistic regression, adjusting for potential confounders identified in univariate analysis. The -2loglikelihood ratios were used to explore the interaction and effect modification of selected variables. Statistical significance was set at *p* < 0.05. 

### 2.4. Ethics Approval and Consent to Participate

All procedures performed in studies involving human participants were in accordance with the ethical standards of the institutional and national research committee and with the 1964 Helsinki declaration and its later amendments or comparable ethical standards. Written informed consent was obtained from all individual participants included in the study. The study has obtained the ethical clearance from the Malaysian Ethics Research Committee (NMRR-16-2468-33734) and the Monash University Human Research Ethics Committee (Project Number = 8104).

## 3. Results

### 3.1. Characteristics of the Participants

Characteristics of the patients including demographics, diabetes related variables, complication rates, and severity are depicted in Table 1. The mean age of the participants was 59.4 ± 8.8 years old. Approximately half (50.7%) of the participants were males. Malays represented the majority of the study participants followed by Indians and Chinese at 63.3%, 26.7%, and 10%, respectively. The mean BMI of the participants was 28.1 ± 4.9 kg/m^2^.

The sample had a mean duration of diabetes and HBa1c of 9.37 years and 7.99%, respectively. Among these participants, 49 (32.7%) were being treated with insulin and oral hypoglycemic agents (OHAs) and 101 (67.3%) with OHAs alone. Nearly three-quarters (73.3%) of participants were classified as inactive based on the International Physical Activity Guidelines used to assess physical activity.

Overall, thirty-four participants had an aDCSI score/complication of zero, while the majority (34.7%) had a score of between two and three. The mean aDCSI score was 1.63 (SD = 1.36). On the basis of the DASS-21 questionnaire, 12% of participants had abnormal (mild-extremely Severe) depression scores while 24% and 8.7% had abnormal anxiety and stress scores, respectively. Nonadherence to their medication regimen was seen among 29% of participants, while the remaining 71% were considered adherent.

### 3.2. Diabetes-Related Variables and QOL

Table 2 presents the result of an independent *t*-test and one-way ANOVA between diabetes-related variables and mean QOL scores. There was a significant difference in mean QOL scores among participants being treated with OHA and insulin as compared with those treated with only OHA (87.2 ± 7.53 vs. 90.5 ± 8.17, *p* = 0.022). A significant association was also observed among participants who were physically active as compared with those who were inactive (91.6 ± 6.01 vs. 88.6 ± 8.57, *p* = 0.045), and HbA1c ≤ 6.5% vs. > 6.5% (93.4 ± 4.53 vs. 88.3.2 ± 8.33, *p* = 0.01). There was no significant difference between duration of diabetes and mean QOL scores.

Among the diabetes complications assessed, participants with retinopathy had significantly lower mean QOL scores as compared with those without retinopathy (87.5 ± 7.74 vs. 90.5 ± 8.34, *p* = 0.03). This was also seen among subjects with neuropathy (88.3 ± 8.74 vs. 91.2 ± 6.51, *p* = 0.031). The difference in the mean QOL score among nephropathy and macrovascular complication categories, however, was not statistically significant.

Overall, participants with abnormal depression, anxiety, and stress levels had significantly lower mean QOL scores than participants with normal levels of these symptoms, i.e., normal and abnormal depression scores (84.06 ± 9.64 vs. 90.15 ± 7.57, *p* = 0.002); normal and abnormal anxiety score (85.6 ± 7.77 vs. 90.6 ± 7.80, p = 0.001), and normal and abnormal stress scores (84.0 ± 10.18 vs. 89.9 ± 7.68, *p* = 0.011). A significant association was found among participants who were adherent and higher mean QOL scores as compared with those who were nonadherent (90.6 ± 6.89 vs. 86.4 ± 9.90, *p* = 0.014). 

### 3.3. Determinants of Quality of Life

Participants’ QOL scores were divided into two subgroups. Those who scored ≤85 points were included in the poor-moderate category while those who scored ≥86 were classified as having a good-excellent QOL. Overall, 41 (37.3%) subjects fell into the poor-moderate QOL category while 109 (72.7%) fell into the good-excellent QOL category. 

Table 3 presents the results of a simple and multiple binary logistic regression to predict good-excellent QOL status among our study participants. The six significant parameters that were included into the final regression model were HbA1c, depression, anxiety, stress, MA, and aDCSI score. 

The T2DM patients with a HbA1c ≤6.5% were 20 times more likely to have a good-excellent QOL as compared with those with a HbA1c >6.5% (aOR = 20.4, *p* = 0.005, 95% CI = 2.5–175.9). The T2DM participants who were adherent to their medication had 3.3 times higher odds of having a good-excellent QOL as compared with those who were nonadherent (aOR = 3.35, *p* = 0.012, 95% CI = 1.3–8.7). The T2DM patients who had normal levels of anxiety were close approximately five and a half times for likely to have a good-excellent QOL (aOR = 5.73, *p* = 0.004, 95% CI = 1.8–18.5). Finally, patients with an aDCSI score of zero and between two and three were 7.8 times (aOR = 7.78, *p* = 0.013, 95% CI = 1.5–39.2), and 8.2 times (aOR = 8.23, *p* = 0.003, 95% CI = 2.1–32.8) more likely to have a good-excellent QOL as compared with T2DM patients with an aDCSI score of greater than or equal to four.

Further analysis was conducted to explore the potential of MA as an interaction variable or an effect modifier of the relationship between other independent variables and QOL (Table 4). The changes in beta coefficient values suggests MA to be an effect modifier of impact of HbA1c, depression, anxiety, and disease severity index on QOL. 

## 4. Discussion

Sociodemographic factors such as age, gender, income, and education level were not associated with QOL in this study. These findings were similarly observed in a recent study by Chew et al. [30] On the contrary, factors such as lower income and lesser education have been linked to a poorer QOL in studies across several other countries [31,32]. The differences may be due to the various factors including affordable healthcare services and a lower cost of living in Malaysia [30].

Our study demonstrated significantly lower QOL scores among patients with depression, anxiety, and stress. Despite these associations, only abnormal anxiety levels were found to be a significant predictor of having a poor-moderate QOL (Table 3). This was unlike other studies that showed all three psychological disorders were predictors of QOL [33,34,35]. Depression and stress may not have been established as significant predictors, due to the small number of participants who had abnormal levels of these symptoms as compared to the anxiety category as determined by the DASS-21 questionnaire. Furthermore, the use of the DASS-21 questionnaire, despite being a useful screening tool, cannot be used to diagnose depression, anxiety, or stress disorders. The use of a diagnostic tool instead to establish a formal diagnosis of mental health disorders may ultimately aid in establishing an accurate association with QOL. 

This study revealed that certain diabetes complications and increasing complication severity were evidently associated with poorer QOL. The presence of retinopathy and neuropathy were significantly associated with lower QOL scores in contrast to participants with nephropathy and macrovascular complications which were not significantly associated with QOL. Our study results conform to the current literature [18,36,37]. The symptomatic nature of retinopathy and neuropathy, as opposed to nephropathy without ESRD, may underlie the difference in impact it has on QOL. Due to the sample size of patients with the presence of macrovascular complications, it would be premature to assume that the lack of association seen in this study may clinically hold true.

An aggregate of diabetes complication severity as established by the aDCSI scores was a significant predictor of good-excellent QOL. Despite a significant association between those with a score of one and between two and three with those with a score greater than or equal to four, there was no association between those with a score of zero as compared to those with a score greater than four. This may be attributed to missed complications that may be present due to poor reporting and documentation. Major diabetes complications with worse severity along with the presence of more complications, however, have been associated with poorer QOL in other studies [36,37]. 

Participants treated with insulin and OHA had significantly poorer QOL scores as compared with those treated with OHAs alone. A study by Shim et al. also observed a similar trend among patients on insulin containing treatment regimens [38]. Complexity and pain from needle pricks are among many reasons insulin use contributes to a poorer QOL. In spite of this, compliance rates were not affected as MA did not differ significantly among subjects on insulin containing and absent regimens. These results were supported by another Malaysian study by Sufiza et al. [39]. In a recent study in Cameroon where the cost of insulin was not subsidized and exceeded the cost of OHAs, there was a significantly lower adherence rate among subjects on insulin containing regimens [40]. This would imply that despite the difficulty and complexity of insulin use and its impact on QOL, affordability was key to establishing adherence. 

Interestingly, this study also showed that MA was an effect modifier of the relationships between HbA1c, depression, anxiety, and disease severity and QOL. These results have similarly been observed by other studies conducted in Malaysia and Indonesia [11,41]. MA has been linked to improvement in QOL through several direct and indirect factors. Perception of disease control is one factor that although not assessed by this study, has been associated with both improved MA and QOL by other studies [42,43]. This relationship highlights the importance of improving perception of disease control among patients through various ways including patient education, which may result in better MA and overall QOL.

Among all the factors that were significantly associated with QOL, having an HbA1c of ≤6.5% was found to be the strongest predictor of having a good-excellent QOL. This association was also noted by a recent study by Goh et al. [19]. HbA1c levels as a predictor may not only be reflective as a cause for better QOL, but also the effect of better QOL. Overall, the high rates of a good-excellent QOL among Malaysia’s T2DM population, as observed in this study, may revolve around medication affordability. Medication affordability would offer patients the choice of a suitable treatment regimen. Establishing adherence through preferred regimens and affordability would lead to better HbA1c control. This improvement in MA and HbA1c control, could translate into a mortality benefit and also establish a morbidity benefit through QOL improvement. 

This study had several limitations. First the cross-sectional design does not allow for any cause and effect or a temporal relationship to be established. In addition, the use of certain instruments that could not objectively assess outcomes, may have influenced the consistency of our results. Furthermore, associations between certain variables may not have been established due to a small sample size that represented these categories. Lastly, the study was only conducted on samples from a single center recruited via convenience sampling, thus limiting the external validity of these results. 

## 5. Conclusions

Among comorbid conditions linked to having a poor QOL, T2DM has been established as an important condition that needs to be addressed. There are several modifiable factors related to having T2DM that have been linked to this group of patients having a poor QOL. We conducted this project in hopes of shedding some light on the possible predictors of QOL and the complex relationships between them. We found that lower diabetes complications and severity, better MA, and psychosocial well-being through direct and indirect ways were important predictors of better QOL among Malaysians with T2DM. Overall, all the factors discovered to be predictors are modifiable and can be prevented among T2DM patients. 

## Figures and Tables

**Table 1:** Characteristics of the study participants (n = 150).

**Demography**	Age (years)	Mean (SD)	59.4 (8.8)
	Sex	Male	76 (50.7)
		Female	74 (49.3)
	Ethnicity	Malay	95 (63.3)
		Chinese	15 (10.0)
		Indian	40 (26.7)
	Education level	Primary	30 (20.0)
		Secondary	72 (48.0)
		Tertiary	48 (32.0)
	Occupation	Employed	68 (45.3)
		Unemployed/retired	82 (54.7)
	Monthly household income (MYR)	<3000	63 (44.0)
		3000–4999	46 (30.7)
		≥5000	38 (25.3)
**Clinical**	Body Mass Index (kg/m2)	Mean (SD)	28.1 (4.9)
	Physical activity	Inactive	110 (73.3)
		Active	40 (26.7)
	Systolic blood pressure (mmHg)	Mean (SD)	139 (16)
	Diastolic blood pressure (mmHg)	Mean (SD)	76 (10)
	Glycated hemoglobin, HbA1c (%)	Mean (SD)	8.0 (1.8)
	Duration of diabetes (years)	Mean (SD)	9.4 (6.0)
	Diabetes medication	OHAs	101 (67.3)
		OHA + Insulin	49 (32.7)
	Hypoglycemia	Never	129 (86.0)
		At least once a month	21 (14.0)
	Medication adherence	Nonadherent	43 (28.7)
**Complication**	Retinopathy	Cataract	17 (11.3)
		Proliferative	25 (16.7)
		Background	13 (8.7)
		None	98 (65.3)
	Nephropathy		57 (38.0)
	Neuropathy		92 (61.3)
	Stroke/TIA		2 (1.3)
	Cardiovascular Disease	Atherosclerosis	17 (11.3)
		Angina Pectoris	1 (0.7)
		Myocardial Infarction	1 (0.7)
		None	132 (88.0)
	aDCSI score	Mean (SD)	1.6 (1.4)
		0	34 (22.7)
		1	46 (30.7)
		2–3	52 (34.7)
		≥4	18 (12.0)
**Psychosocial well-being**	Depression		18 (12.0)
	Anxiety		36 (24.0)
	Stress		12 (8.0)

Abbreviations: OHAs, oral hypoglycemic agents; aDCSI, adapted diabetes complications and severity index; TIA, transient ischemic attack. Data presented as n (%) unless indicated.

**Table 2:** Comparison of QOL scores according to characteristics of the study participants (n = 150).

Variables	QOL Scores Mean (SD)	Mean Difference (95% CI)	*p*
Age (years)			
<50 ^a^	88.6 (11.80)		0.582
50–59	88.56 (7.65)	−0.09 (5.53, −5.71)	
60–69	90.57 (7.65)	1.92 (7.55, −3.71)	
>70	89.41 (5.03)	0.76 (7.90, −6.37)	
Ethnicity			
Malay ^a^	89.86 (8.07)		0.655
Indian	88.48 (8.41)	−0.73 (4.71, −6.17)	
Chinese	89.13 (7.23)	−0.14 (2.30, −5.08)	
Gender ^b^			
Male	88.8 (8.76)	−1.23 (-3.83, 1.38)	0.354
Female	90.04 (7.28)		
Education Level			
Primary ^a^	89.63 (4.51)		0.894
Secondary	89.10 (8.42)	−0.54 (3.73, 4.80)	
Tertiary	89.77 (9.27)	0.14 (4.71, −4.43)	
Monthly Household Income (MYR)			
≤1000 ^a^	84.29 (7.30)		0.469
1001–2000	88.22 (8.91)	3.93 (14.33, −6.47)	
2001–3000	89.36 (7.65)	5.08 (15.03, −4.88)	
3001–4000	89.36 (7.12)	5.08 (15.53, −5.37)	
4001–5000	90.00 (7.75)	5.71 (16.06, −4.63)	
>5000	89.42 (8.06)	6.53 (15.44, −3.38)	
Diabetes duration (years)			
< 5 ^a^	89.57 (8.18)		0.100
5–10	91.23 (6.48)	1.67 (5.72, −2.39)	
>10	87.86 (8.88)	−1.70 (2.15, −5.56)	
Type of treatment ^b^			
OHAs	90.46 (8.17)	3.23 (0.46, 5.99)	0.022 *
OHAs and insulin	87.23 (7.53)		
HbA1c			
<6.5 ^a^	93.39 (4.50)		0.021 *
6.5–7.5	90.17 (8.31)	−3.22 (2.05, −8.49)	
7.6–8.5	87.5 (7.76)	−5.89 (−0.20, −11.5)	
>8.5	87.82 (8.05)	−5.56 (−0.08, −11.05)	
Physical activity ^b^			
Active	91.6 (6.01)	2.97 (5.88, 0.061)	0.045 *
Inactive	88.6 (8.57)		
Retinopathy ^b^			
Yes	87.46 (7.74)	−3.00 (−5.70, −0.30)	0.030 *
No	90.46 (8.34)		
Nephropathy ^b^			
Yes	88.82(8.57)	−0.96 (−3.64, 1.72)	0.481
No	89.78 (7.95)		
Neuropathy ^b^			
Yes	88.29 (8.74)	−2.91 (−5.55, −0.27)	0.031 *
No	91.20 (6.51)		
Macrovascular Complications ^b^			
Yes	86.9(8.47)	−2.91 (−6.72, 0.90)	0.134
No	83.7 (10.76)		
Hypoglycemia ^b^			
Never	90.3 (7.16)	−6.63 (−1.61, −11.66)	0.012 *
At least once/month	86.3 (7.88)		
aDCSI categories			
0	92.26 (6.03)		0.001 **
1	87.15 (8.61)	−5.11 (−9.60, −0.62)	
2–3	91.88(6.08)	−0.04 (−4.76, 4.00)	
4	82.72 (9.76)	−9.54 (−15.33, −3.76)	
Depression ^b^			
Yes	84.06 (9.64)	6.10 (2.20, 9.99)	0.002 *
No	90.15 (7.57)		
Anxiety ^b^			
Yes	85.6 (7.77)	5.04 (2.11, 7.99)	0.001 **
No	90.6 (7.80)		
Stress ^b^			
Yes	84.0 (10.18)	5.93 (1.40, 10.47)	0.011 *
No	89.9 (7.68)		
Medication adherence ^b^			
Yes	90.6(6.89)	4.17 (0.87,7.47)	0.014 *
No	86.4, (9.90)		

Notation: post hoc Bonferroni ^a^ reference group, ^b^ independent *t*-test, * significant at *p* < 0.05, ** significant at *p* < 0.001.

**Table 3:** Simple and multiple binary logistic regression analysis of factors associated with quality of life of study participants (n = 150).

Factors	n (%)		c0R (95% CI)	*p*	aOR (95%CI)	*p*
	Poor-Moderate QOL	Good-Excellent QOL				
Hba1c						
≤6.5%	1 (3.2)	30 (96.8)	15.19 (2.00, 115.47)	0.009 *	20.78 (2.45, 175.94)	0.005 *
>6.5%	40 (33.6)	79 (66.4)	1.00		1.00	
Depression						
Yes	7 (38.9)	11 (61.1)	1.83 (0.66, 5.11)	0.246	0.63 (0.13, 3.10)	0.568
No	34 (25.8)	98 (74.2)	1.00		1.00	
Stress						
Yes	5 (38.5)	8 (61.5)	1.75 (0.54, 5.71)	0.351		
No	36 (26.3)	101 (73.7)	1.00			
Anxiety						
Yes	16 (44.4)	20 (55.6)	2.85 (1.29,6.30)	0.01 *	5.73 (1.77, 18.52)	0.004 *
No	25 (21.9)	89 (78.1)	1.00		1.00	
Medication Adherence						
Yes	22 (20.6)	85 (79.4)	3.06 (1.43, 6.56)	0.004 *	3.35 (1.30, 8.66)	0.012 *
No	19 (44.2)	24 (55.8)	1.00		1.00	
aDCSI						
0	4 (11.8)	30 (88.2)	9.38 (2.32, 37.92)	0.002 *	7.78 (1.54, 39.15)	0.013 *
1	19 (41.3)	27 (58.7)	1.78 (0.59, 5.33)	0.306	1.51 (0.41, 5.52)	0.536
2–3	8 (44.4)	10 (55.6)	6.88 (2.08, 22.75)	0.002 *	8.23 (2.06, 32.84)	0.003 *
≥4	10 (55.6)	8(44.4)	1.00		1.00	

Notation: The enter method in the multiple logistic regression method was applied. No multicollinearity was detected. The Hosmer–Lemeshow test (*p* = 0.658), classification table (overall correctly classified = 80.7%) and area under the ROC curve (87.5%) was applied to test the model fitness. Abbreviations: c0R, crude odds ratio and aOR, adjusted odds ratio. * significant at *p* < 0.05, ** significant at *p* < 0.05.

**Table 4:** Interaction and effect modification of medication adherence.

Model		-2loglikelihood	Deviance	Model Significance ^a^	Change in β ^b^
1	Full model	126.788	49.176	*p* < 0.05	-
2	Full model + HbA1c*MA	126.049	0.739	*p* > 0.05	595.22%
3	Full model + Depression*MA	125.556	1.232	*p* > 0.05	24.52%
4	Full model + Anxiety*MA	125.758	1.030	*p* > 0.05	62.92%
5	Full model + aDCSI*MA	124.857	1.931	*p* > 0.05	0: 85.57% 1: 310.49% 2–3: 31.02%

Notation: ^a^ not significant model (*p* > 0.05) indicates MA was not an interaction variable, while ^b^ changes of coeffcient value of >15% indicates MA to be an effect modifier.

## References

[1] Matthews D., McDowell J., Matthews D.M., Brown F.J. (2007). What is diabetes?. Diabetes—A Handbook for the Primary Healthcare Team.

[2] International Diabetes Federation IDF Diabetes Atlas Diabetes Facts and Figures. http://www.idf.org/about-diabetes/facts-figures.

[3] NHMS I. (1986). The First National Health and Morbidity Survey (NHMS I) 1986.

[4] NHMS (2015). National Health and Morbidity Survey (NMHS 2015): Non-Communicable Diseases, Risk Factors and Other Health Problems.

[5] UK Prospective Diabetes Study Group (1998). Tight blood pressure control and risk of macrovascular and microvascular complications in type 2 diabetes. BMJ.

[6] Odegard P.S., Capoccia K. (2007). Medication taking and diabetes: A systematic review of the literature. Diabetes Educ..

[7] Rubin R.R. (2005). Adherence to pharmacologic therapy in patients with type 2 diabetes mellitus. Am. J. Med..

[8] Asche C., LaFleur J., Conner C. (2011). A review of diabetes treatment adherence and the association with clinical and economic outcomes. Clin. Ther..

[9] Martínez Y.V., Prado-Aguilar C.A., Rascón-Pacheco R.A., Valdivia-Martínez J.J. (2008). Quality of life associated with treatment adherence in patients with type 2 diabetes: A cross-sectional study. BMC Health Serv. Res..

[10] Piette J.D., Wagner T.H., Potter M.B., Schillinger D. (2004). Health insurance status, cost-related medication underuse, and outcomes among diabetes patients in three systems of care. Med. Care.

[11] Chew B.H., Hassan N.H., Sherina M.S. (2015). Determinants of medication adherence among adults with type 2 diabetes mellitus in three Malaysian public health clinics: A cross-sectional study. Patient Prefer. Adherence.

[12] Chew B.H. (2015). Medication adherence on quality of life among adults with type 2 diabetes mellitus: An exploratory analysis on the EDDMQoL study. Qual. Life Res..

[13] Snoek F.J., Skinner T.C. (2006). Psychological aspects of diabetes management. Medicine.

[14] Lin E.H., Von Korff M., Alonso J., Angermeyer M.C., Anthony J., Bromet E., Bruffaerts R., Gasquet I., de Girolamo G., Gureje O. (2008). Mental disorders among persons with diabetes--results from the World Mental Health Surveys. J. Psychosom Res..

[15] Leal J., Gray A.M., Clarke P.M. (2009). Development of life-expectancy tables for people with type 2 diabetes. Eur. Heart J..

[16] Rubin R.R., Peyrot M. (1999). Quality of life and diabetes. Diabetes Metab. Res. Rev..

[17] De Visser C.L., Bilo H.J.G., Groenier K.H., De Visser W., Meyboom-De Jong B. (2002). The influence of cardiovascular disease on quality of life in type 2 diabetics. Qual. Life Res..

[18] UK Prospective Diabetes Study Group (1999). Quality of life in type 2 diabetic patients is affected by complications but not by intensive policies to improve blood glucose or blood pressure control (UKPDS 37). Diabetes Care.

[19] Goh S.G., Rusli B.N., Khalid B.A. (2015). Diabetes quality of life perception in a multiethnic population. Qual. Life Res..

[20] Egede L.E., Hernandez-Tejada M.A. (2013). Effect of comorbid depression on quality of life in adults with Type 2 diabetes. Expert Rev. Pharm. Outcomes Res..

[21] Eren I., Erdi Ö., Şahin M. (2008). The effect of depression on quality of life of patients with type II diabetes mellitus. Depress. Anxiety.

[22] Goh S.G., Rusli B.N., Khalid B.A. (2015). Development and validation of the Asian Diabetes Quality of Life (AsianDQOL) Questionnaire. Diabetes Res. Clin. Pract..

[23] Chang H.Y., Weiner J.P., Richards T.M., Bleich S.N., Segal J.B. (2012). Validating the adapted Diabetes Complications Severity Index in claims data. Am. J. Manag. Care.

[24] Chung W.W., Chua S.S., Lai P.S.M., Morisky D.E. (2015). The Malaysian Medication Adherence Scale (MALMAS): Concurrent validity using a clinical measure among people with type 2 diabetes in Malaysia. PLoS ONE.

[25] Lovibond P.F., Lovibond S.H. (1995). The structure of negative emotional states: Comparison of the Depression Anxiety Stress Scales (DASS) with the Beck Depression and Anxiety Inventories. Behav. Res. Ther..

[26] Davies G., Caputi P., Skarvelis M., Ronan N. (2015). The depression anxiety and stress scales: Reference data from a large psychiatric outpatient sample. Aust. J. Psychol..

[27] Musa R., Fadzil M.A., Zain Z. (2007). Translation, validation and psychometric properties of Bahasa Malaysia version of the Depression Anxiety and Stress Scales (DASS). ASEAN J. Psychiatr..

[28] Green S.B. (1991). How many subjects does it take to do a regression analysis. Multivar. Behav. Res..

[29] Craig C.L., Marshall A.L., Sjostrom M., Bauman A.E., Booth M.L., Ainsworth B.E., Barbara E., Pratt M., Ekelund U., Yngve A. (2003). International physical activity questionnaire: 12-country reliability and validity. Med. Sci. Sports Exerc..

[30] Chew B.H., Mohd-Sidik S., Shariff-Ghazali S. (2015). Negative effects of diabetes-related distress on health-related quality of life: An evaluation among the adult patients with type 2 diabetes mellitus in three primary healthcare clinics in Malaysia. Health Qual. Life Outcomes.

[31] Glasgow R.E., Ruggiero L., Eakin E.G., Dryfoos J., Chobanian L. (1997). Quality of life and associated characteristics in large national sample of adults with diabetes. Diabetes Care.

[32] Didarloo A., Alizadeh M. (2016). Health-related quality of life and its determinants among women with diabetes mellitus: A cross-sectional analysis. Nurs. Midwifery Stud..

[33] Mosaku K., Kolawole B., Mume C., Ikem R. (2008). Depression, anxiety and quality of life among diabetic patients: A comparative study. JAMA.

[34] Kohen D., Burgess A.P., Catalan J., Lant A. (1998). The role of anxiety and depression in quality of life and symptom reporting in people with diabetes mellitus. Qual. Life Res..

[35] Dos Santos M.A.B., Ceretta L.B., Réus G.Z., Abelaira H.M., Jornada L.K., Schwalm M.T., Neotti M.V., Tomazzi C.D., Gulbis K.G., Ceretta R.A. (2014). Anxiety disorders are associated with quality of life impairment in patients with insulin-dependent type 2 diabetes: A case-control study. Braz. J. Psychiatry.

[36] Coffey J.T., Brandle M., Zhou H., Marriott D., Burke R., Tabaei B.P., Engelgau M.M., Kaplan R.M., Herman W.H. (2002). Valuing health-related quality of life in diabetes. Diabetes Care.

[37] Alcubierre N., Rubinat E., Traveset A., Martinez-Alonso M., Hernandez M., Jurjo C., Muricio D. (2014). A prospective cross-sectional study on quality of life and treatment satisfaction in type 2 diabetic patients with retinopathy without other major late diabetic complications. Health Qual. Life Outcomes.

[38] Shim Y.T., Lee J., Toh M.P.H.S., Tang W.E., Ko Y. (2012). Health-related quality of life and glycaemic. control in patients with Type 2 diabetes mellitus in Singapore. Diabet. Med..

[39] Sufiza Ahmad N., Ramli A., Islahudin F., Paraidathathu T. (2013). Medication adherence in patients with type 2 diabetes mellitus treated at primary health clinics in Malaysia. Patient Prefer. Adherence.

[40] Aminde L.N., Tindong M., Ngwasiri C.A., Aminde J.A., Njim T., Fondong A.A., Fondong A.A., Takah N.F. (2019). Adherence to antidiabetic medication and factors associated with non-adherence among patients with type-2 diabetes mellitus in two regional hospitals in Cameroon. BMC Endocr. Disord..

[41] Alfian S.D., Sukandar H., Lestari K., Abdulah R. (2016). Medication adherence contributes to an improved quality of life in type 2 diabetes mellitus patients: A cross-sectional study. Diabetes Ther..

[42] Gonzalez J.S., Shreck E., Psaros C., Safren S.A. (2015). Distress and type 2 diabetes-treatment adherence: A mediating role for perceived control. Health Psychol..

[43] Hernandez-Tejada M.A., Lynch C.P., Strom J.L., Egede L.E. (2012). Effect of perceived control on quality of life in indigent adults with type 2 diabetes. Diabetes Educ..

